# *Qing-Yi decoction* in participants with severe acute pancreatitis: a randomized controlled trial

**DOI:** 10.1186/s13020-015-0039-8

**Published:** 2015-05-19

**Authors:** Weiwei Chen, Xiaonan Yang, Lei Huang, Ping Xue, Meihua Wan, Jia Guo, Lin Zhu, Tao Jin, Zongwen Huang, Guangyuan Chen, Wenfu Tang, Qing Xia

**Affiliations:** Department of Integrated Traditional Chinese and Western Medicine, West China Hospital, Sichuan University, Chengdu, 610041 Sichuan Province China

## Abstract

**Background:**

*Qing-Yi Decoction* (QYD) has been used for severe acute pancreatitis (SAP) patients in China for many years. There were two kinds of QYD: Num 1. QYD (QYD1) which is used in the acute response stage of SAP and Num 2. QYD (QYD2) which is used in the second stage of SAP. This study aims to evaluate the therapeutic efficacy of QYD in participants with SAP.

**Methods:**

In this prospective, randomized, double-blind, placebo-controlled trial, participants aged 18–70 years within the first 7 days after acute onset of typical abdominal pain (the definition of SAP was according to the 2007 Guidelines for Management of Severe Acute Pancreatitis in China) were selected. The disease severity was determined by the Ranson, Acute Physiology and Chronic Health Evaluation II, and Balthazar CT scores. The test group received Western medicine and Chinese medicine (Num.1 QYD and Num.2 QYD), while the control group received Western medicine and placebo. The primary end-points were length of hospital stay, total hospitalization expenses, operation rate, and mortality. The secondary end-points were organ complications (*i.e.*, heart failure, respiratory failure, acute renal failure, and hepatic failure), duration of paralytic ileus, infection, intensive care unit stay, and respirator use.

**Results:**

From March 2008 to July 2010, a total of 300 participants with severe acute pancreatitis were assessed for eligibility in West China Hospital, and 100 were eligible for randomized allocation. Eighty-five participants (46 in the test group; 39 in the control group) were included in the statistical analyses. The two groups were similar in their baseline clinical characteristics (age, sex, and etiology) and disease severity. After the interventions, there were no differences between the two groups for length of hospital stay (*P* = 0.323), total hospitalization expenses (*P* = 0.252), operation rate (*P* = 0.231), mortality (*P* = 0.462), organ complications (*P* > 0.05), intensive care unit stay (*P* = 0.209), and respirator use (*P* > 0.05). However, the duration [median (interquartile range)] of paralytic ileus, *i.e.*, 4 (2–6) days *vs.* 6 (4–8) days (*P* = 0.014) and rate of infection, *i.e.*, (13.0 % *vs.* 35.9 %) (*P* = 0.013) differed significantly.

**Conclusions:**

QYD could restore gastrointestinal motility to normal and reduce the infection rates in the SAP patients who completed a full course of QYD treatment according to per protocol analysis.

**Electronic supplementary material:**

The online version of this article (doi:10.1186/s13020-015-0039-8) contains supplementary material, which is available to authorized users.

## Background

The overall mortality rates of severe acute pancreatitis (SAP) are approximately 15–30 % in worldwide [1], and 11.8–16.3 % in China [2, 3]. In patients with SAP, early deaths primarily result from multisystem organ failure, while late deaths primarily arise through complications in patients with infected necrosis [4, 5]. Clinically, gastrointestinal motility disorders, such as paralytic ileus, abdominal pain, distension, and even intra-abdominal hypertension and abdominal compartment syndrome, commonly encountered in the first stage of SAP are the triggers for multiple organ failure [6]. Based on observations in pancreatitis models in animals, bacterial overgrowth and bacterial translocation were involved in the pathogenesis of pancreatitis-induced sepsis [7]. Chinese medicine (CM) active ingredients, such as emodin (*Dahuangsu*), magnolol (*Houpufen*), naringin (*Youpigan*), ginkgolide B (*Yinxingneizhi*), sanchinoside (*Sanqizaogan*), taxol (*Zishanfen*), resveratrol (*Baililuchun*), rutoside (*Yunxianggan*), tetramethylpyrazine (*Chuanxiongqin*), and breviscapine (*Dengzhanhuasu*), being used in treating SAP have advantages in not only acting on the pancreas, stomach, and intestines, but also having marked treatment effects on other visceral injuries arising from systemic inflammatory responses accompanying pancreatitis and blocking the disease progression [8].

CM has been used for the treatment of SAP patients in China for several decades [9, 10]. In data collected from West China Hospital for 1561 SAP patients between 1980 and 2005, it was found that patients who received a combination of CM and Western medicine treatment had an overall mortality of <10.25 % from 1994 to 2005 [11]. In integrated CM and Western medicine therapy, the *Chai-Qin-Cheng-Qi Decoction* (CQCQD), which is modified from the *Da-Cheng-Qi Decoction* (DCQD) (*Cortex Magnoliae Officinalis (Houpu)*, *Fructus Aurantii Immaturus (Zhike)*, *Radix et Rhizoma Rhei (Dahuang)*, and *Natrii Sulfas (Mangxiao)*), is widely used for acute pancreatitis (AP) patients in China [12]. In the acute response stage, both DCQD and CQCQD promoted the recovery of intestinal mucosal permeability [13], improved intestinal paralysis, reduced intra-abdominal hypertension and length of hospital stay [14], shortened the duration of gastrointestinal failure and secondary infections [15], reduced AP-associated lung injury [16–18], improved gastric dysrhythmia, enhanced gastrointestinal motility, adjusted the synchronized recovery of the alimentary tract, and increased plasma ghrelin after abdominal surgery [19].

*Qing-Yi Decoction* (QYD) also modified from DCQD has been used for SAP patients in China for many years. Many Chinese studies showed that QYD can inhibit the release of TNF-α and IL-8 to reduce inflammation of rats with SAP [20, 21], was effective in treating experimental SAP involved the MAPK and NLR signaling pathways, cell cycle, metabolic pathways, and oxide reductase activities [22]. QYD has two types: Num.1 *Qing-Yi decoction* (QYD1) is usually used in the acute response stage of SAP patients for purgating *heat* and bowels, dispersing stagnated *liver qi*; And Num.2 *Qing-Yi decoction* (QYD2) is used in the second stage mainly for clearing away *heat* and *toxic* material, promoting *blood* circulation for removing *blood stasis*. Facing the same disease, these two kinds of decoction focus on the different emphases according to the different stages.

However, few prospective randomized controlled trials (RCT) have been designed to determine the efficacy of QYD. This study aims to conduct an RCT to evaluate the therapeutic efficacy of QYD in treating SAP.

## Methods

### Study approval and registration

According to the Declaration of Helsinki and Good Clinical Practice guidelines [23], the protocol was reviewed and approved by the West China Hospital Ethics Committee, Sichuan University [2008(12)] (Additional files 1 and 2). This trial was registered in the Chinese Clinical Trials Register (Registration number: ChiCTR-TRC-14004403). We reported the results in accordance with the Consolidated Standards of Reporting Trials statement [24].

### Randomization and allocation concealment

Randomization sequence was performed with SAS software (SAS Institute, USA) by two statisticians who were not involved in the contact with participants. Sequentially numbered sealed opaque envelopes containing allocation codes were produced for allocation concealment by coordinator in a separate location at the West China Hospital. After recruitment of each participant and before admission to any group, a numbered envelope was opened at the location and a card inside only showed if the participant was to be in group A or group B. This information was then given to the investigators. The coordinator was not involved in the recruitment process for the study. The recruitment investigators were not involved in the randomization and allocation concealment process and were blinded to the types of treatment received by the participants. The participants involved also remained unaware of the actual medications administered during the entire study period.

### Selection of participants

This trial was conducted at West China Hospital of Sichuan University (one of the largest treatment centers for SAP in China) between March 2008 and July 2010. Three hundred potential participants were contacted. They or their representatives understood the study. A total of 236 participants provided written medical informed consent prior to the clinical procedures (Additional file 3). All participants were recruited and examined by investigators according to the inclusion and exclusion criteria described below.

The diagnosis of SAP was established on the basis of acute onset of typical abdominal pain with at least three times the upper limit of the reference range for serum amylase or lipase, ultrasonographic or computerized tomographic (CT) scan evidence of pancreatitis, and an Acute Physiology and Chronic Health Evaluation II (APACHE II) score of ≥8, or a Balthazar CT score of ≥4, or a Ranson score of ≥3 [25].

The inclusion criteria were: (a) all hospitalized adult participants aged 18–70 years; (b) confirmed diagnosis of SAP according to the 2007 Guidelines for Management of Severe Acute Pancreatitis in China [25]; and (c) participants within the first 7 days after acute onset of typical abdominal pain.

The exclusion criteria were: (a) pregnant or lactating women; (b) participants with malignancy; (c) participants in a pre-death state (terminal phase of other diseases); or (d) participants who had received other CM treatment prior to hospital admission.

Participants recruited into the trial were withdrawn if they were lost to follow-up, refused enemas, dropped out, or had serious adverse effects.

### Clinical treatment

According to the 2007 Guidelines for Management of Severe Acute Pancreatitis in China [25] and depending on the disease severity and individual situations, two groups were treated with the same Western medicine treatment regimen, including fasting in the first several days when participants still had severe abdominal pain, gastrointestinal decompression, antacids, intensive organ function monitoring, fluid resuscitation, maintenance of water, electrolyte and acid–base balances, nutritional supplements, and parenteral antibiotics (such as carbapenems, quinolones, and metronidazole) for suspected infections with microbiological evidence or clinical diagnosis. Assisted mechanical ventilation and intensive care were performed if necessary. Participants with suspected pancreatic infections were evaluated by an experienced pancreatic surgeon. In addition, the participants in the test group received CM therapy comprising QYD1 and QYD2, while the participants in the control group received placebo formulas made of dextrin that were designed to resemble the QYD1 and QYD2 formulas in their packaging, form, color, taste, size, and smell.

QYD1 was composed of *Chaihu* (*Radix Bupleuri*), 9 g; *Huangqin* (*Radix Scutellariae*), 9 g; *Chuanjizi* (*Fructus Toosendan*), 9 g; *Yuanhu* (*Rhizoma Corydalis*), 9 g; *Dahuang* (*Radix et Rhizoma Rhei*), 15 g; *Mudanpi* (*Cortex Moutan*), 9 g; *Zhishi* (*Fructus Aurantii Immaturus*), 9 g; *Houpo* (*Cortex Magnoliae Officinalis*), 9 g; *Mangxiao* (*Natrii Sulfas*), 10 g; and *Gansui* (*Radix Kansui*), 0.2 g.

QYD2 was composed of *Chaihu* (*Radix Bupleuri*), 9 g; *Huangqin* (*Radix Scutellariae*), 9 g; *Chuanjizi* (*Fructus Toosendan*), 9 g; *Yuanhu* (*Rhizoma Corydalis*), 9 g; *Dahuang* (*Radix et Rhizoma Rhei*), 30 g; *Mudanpi* (*Cortex Moutan*), 15 g; *Hongteng* (*Caulis Sargentodoxae*), 15 g; *Baijiangcao* (*Herba Patriniae*), 15 g; *Pugongying* (*Herba Taraxaci*), 30 g; *Zihuadiding* (*Herba Violae*), 30 g; and *Taoren* (*Semen Persicae*), 9 g.

Schedule of administration in the test group: In the acute response stage, each batch of QYD1 formula was divided into two packages of granules weighing 10 g. Each package of granules was dissolved in 200 mL of water, and administered orally or intragastrically. After 1 h, two packages were dissolved in 400 mL of water for enema performance. This administration was performed every 6 h daily until the participants’ bowel sound returned to normal. When the participants’ abdominal pain disappeared, oral feeding was encouraged. When participants were able to resume oral feeding, each batch of QYD2 formula was divided into two packages of granules weighing 10 g. Each package of granules was dissolved in 200 mL of water, and administered orally three times daily rather than as an enema.

Schedule of administration in the control group: The two placebo formulas were administered orally, intragastrically, or as an enema in the same manner as the QYD1 and QYD2 formulas in the test group. All prescribed medications used in the two groups were prepared and dispensed by an investigator in a separate room and not involved in contact with participants.

### Outcome measurements

The primary end-points were the length of hospital stay, total hospitalization expenses, operation rate, and mortality. The secondary end-points were organ complications [heart failure, respiratory failure, acute renal failure (ARF), hepatic failure], incidence and duration of paralytic ileus, infection (pneumonia or pancreatic infection diagnosed by microbiologic assessments), intensive care unit (ICU) stay, and respirator use.

### Monitoring and follow-up

All adverse events and compliance with the treatment were recorded by a safety inspector throughout the study. Clinical laboratory indexes, including routine blood, urine, and stool tests, hepatic and renal functions, and electrocardiograms, were undertaken at baseline and once per week after initiation of the treatment to assess the safety for each group. If any emergencies occurred, the physician investigators were notified to deal with them. After completion of the study, a minimum 1-month periodic outpatient follow-up evaluation was performed by related questionnaires (Additional file 4).

### Statistical analysis

All data were analyzed using SAS statistical software (SAS Institute, USA). All quantitative data were expressed as mean and standard deviation (SD), percentage, or median and interquartile range. A two-independent-sample *t*-test was used to compare normally distributed data, while the Wilcoxon rank sum test was applied for non-normally distributed data. The Chi-square test was performed to analyze categorical data. Values of *P* < 0.05 were considered statistically significant. Intention-to-treat (ITT) (Additional file 5) and per-protocol analyses were both performed. The two results were similar. As we intended to exclude other factors and mainly focused on evaluating the actual therapeutic efficacy of QYD instead of its safety, finally per-protocol analysis was reported.

## Results

### Demographic data and baseline characteristics

**Fig. 1:**
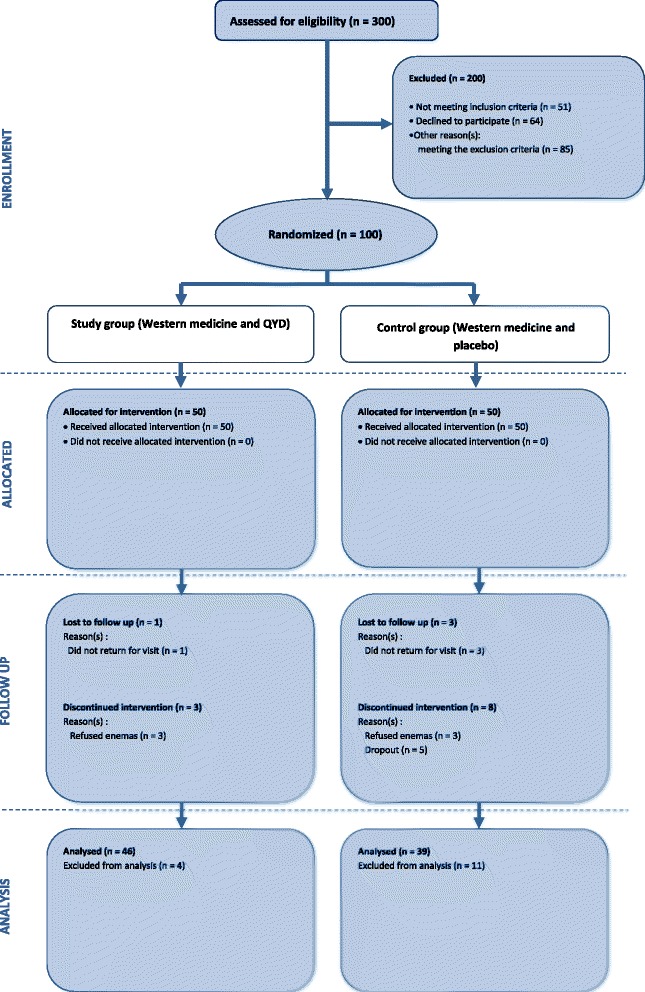
Flowchart of enrolled and randomized participants

A total of 300 participants with SAP were assessed for eligibility from March 2008 to July 2010. Of these participants, 51 did not match the inclusion criteria (21 did not meet the age limitation; 30 were beyond the first 7 days after acute onset of typical abdominal pain), 64 did not agree to participate in the trial, and 85 met the exclusion criteria. In total, 100 participants were eligible for randomization (50 in the test group; 50 in the control group). Four participants were withdrawn from the test group, because one was lost to follow up and three refused to undergo enemas. Additionally, 11 participants were withdrawn from the control group, because three were lost to follow up, three refused to undergo enemas, and five dropped out. Finally, there were 46 participants eligible for analysis in the test group and 39 participants in the control group (Fig. 1).

The participants’ demographics and baseline clinical characteristics in the two groups were summarized in Table 1. The mean ages (SD) were 46.4 (11.3) years in the test group and 44.6 (9.8) years in the control group. The two groups had similar sex distributions. Hyperlipidemia was the most common etiology (56.5 % in the test group *vs.* 38.5 % in the control group), followed by gallstones, alcohol, and idiopathic factors in each group. The Ranson scores [median (interquartile range)] in the test group and control group were 3 (3–5) and 4 (3–5), respectively. The APACHE II scores [median (interquartile range)] evaluated within 24 h of admission were 10 (7.8–13) and 7 (5–13) in the test group and control group, respectively. The Balthazar CT scores [median (interquartile range)] were both 4 (4–6) in the test group and control group. There were no differences in the baseline characteristics in terms of age, sex, and etiology between the two groups (*P* = 0.434, 0.805, 0.201, respectively), or in the Ranson score (*P* = 0.608), 24-h APACHE II score (*P* = 0.150), and Balthazar CT score (*P* = 0.118) in the initial stage of administration.

**Table 1:** Participant demographics and baseline clinical characteristics in the two groups

	Test group (*n* = 46)	Control group (*n* = 39)	*P* value
Age (mean ± SD)^†^			0.434
Years	46.4 (11.3)	44.6 (9.8)	
Sex, *n* (%)^‡^			0.805
Male	26 (56.5)	21 (53.8)	
Female	20 (43.5)	18 (46.2)	
Etiology, *n* (%)^‡^			0.201
Biliary	10 (21.7)	8 (20.5)	
Hyperlipidemic	26 (56.5)	15 (38.5)	
Alcoholic	4 (8.7)	4 (10.2)	
Idiopathic	6 (13.1)	12 (30.8)	
Scores (median (interquartile range))^§^			
Ranson’s score	3 (3–5)	4 (3–5)	0.608
24 h APACHE II score	10 (7.8-13)	7 (5–13)	0.150
Balthazar CT score	4 (4–6)	4 (4–6)	0.118

^†^Two independent samples *t*-test, ^‡^Chi-square test, ^§^Wilcoxon rank sum test

*APACHE II* Acute Physiology and Chronic Health Evaluation II

### Responses to treatment

#### Primary end-points

The comparisons of the primary end-points between the two groups were shown in Table 2. The duration of hospital stay [median (interquartile range)] in the two groups did not differ significantly (*P* = 0.323), comprising 20 (14.8–27.3) days in the test group and 23 (16–28) days in the control group. The total hospitalization expenses [median (interquartile range)] did not differ significantly between the two groups, being RMB33390.1 (24311.7–45996.1) and RMB41523.1 (23216.0–68579.1) in the test group and control group, respectively (*P* = 0.252). There were no significant differences between the two groups for the incidence of surgery (2.2 % *vs.* 7.7 %) (*P* = 0.231) or mortality (2.2 % *vs.* 5.1 %) (*P* = 0.462).

**Table 2:** The primary end points in the two groups

	Test group (*n* = 46)	Control group (*n* = 39)	*P* value
Hospital stay (days; median (interquartile range)^§^)	20 (14.8-27.3)	23 (16–28)	0.323
Total hospitalization expenses (RMB; median (interquartile range)^§^)	33390.1 (24311.7-45996.1)	41523.1 (23216–68579.1)	0.252
Operation, *n* (%)^‡^	1(2.2)	3(7.7)	0.231
Mortality, *n* (%)^‡^	1(2.2)	2(5.1)	0.462

^‡^Chi-square test, ^§^Wilcoxon rank sum test

#### Secondary end-points

The comparisons of the secondary end-points between the two groups were shown in Table 3. The two groups showed no differences in the incidence and duration of organ complications (*P* > 0.05), of which respiratory failure was the most prevalent complication, followed by hepatic failure, heart failure, and ARF. There was also no significant difference in the incidence of paralytic ileus (*P* = 0.157): 95.7 % (44/46) in the test group and 87.2 % (34/39) in the control group. Meanwhile, the durations of paralytic ileus [median (interquartile range)] were 4 (2–6) days in the test group and 6 (4–8) days in the control group. The durations of ICU stay were both 0 (0–0) days in the test group and in the control group. The respirator usage rates were 8.7 % (4/46) and 23.1 % (9/39) in the test group and control group, respectively. The infection rates were 13 % and 35.9 % in the test group and control group, respectively. Both the duration of paralytic ileus (*P* = 0.014) and the rate of infection (*P* = 0.013) showed significant differences between the two groups.

**Table 3:** The secondary end points in the two groups

	Test group (*n* = 46)	Control group (*n* = 39)	*P* value
Organ complications			
Heart failure, *n* (%)^‡^	12 (26.1)	12 (30.8)	0.633
Duration of heart failure, days median (interquartile range)^§^	0 (0–1)	0 (0–2)	0.449
Respiratory failure, *n* (%)^‡^	28 (60.9)	20 (51.3)	0.374
Duration of respiratory failure, days median (interquartile range)^§^	1 (0–3)	1 (0–3)	0.835
ARF, *n* (%)^‡^	5 (10.9)	3 (7.7)	0.617
Duration of ARF, days median (interquartile range)^§^	0 (0–0)	0 (0–0)	0.715
Hepatic failure, *n* (%)^‡^	11 (23.9)	15 (38.5)	0.147
Duration of hepatic failure, days median (interquartile range)^§^	0 (0–0.3)	0 (0–4)	0.069
Paralytic ileus, *n* (%)^‡^	44 (95.7)	34 (87.2)	0.157
Duration of paralytic ileus, days median (interquartile range)^§^	4 (2–6)^a^	6 (4–8)	0.014
Infection, *n* (%)^‡^	6 (13.0)^a^	14 (35.9)	0.013
ICU stay, days median (interquartile range)^§^	0 (0–0)	0 (0–0)	0.209
Use of respirator, *n* (%)^‡^	4 (8.7)	9 (23.1)	0.066
Use of respirator, days median (interquartile range)^§^	0 (0–0)	0 (0–0)	0.067

^‡^Chi-square test, ^§^Wilcoxon rank sum test. ^a^ *P* < 0.05 *vs.* control group

*ARF* acute renal failure

*ICU* intensive care unit

### Safety

One participant died in the test group and two participants died in the control group because of the severity of SAP (discussed below). Diarrhea was the main adverse event in this study, especially after taking QYD1 orally and for enemas in the acute response stage. However, diarrhea was also the main therapeutic pathway for SAP patients to recover gastrointestinal function in CM. Antidiarrheal agents were not allowed, as the maintenance of water, electrolyte, and acid–base balances was of great importance in this situation. No other adverse events were reported in the two groups. In addition, no significant changes were recorded in the laboratory indexes or physical examinations.

## Discussion

The participants with SAP in the test group receiving the QYD1 and QYD2 formulas demonstrated significant improvements in the duration of paralytic ileus and the rate of infections compared with the participants receiving the placebo formulas in the control group. The other outcomes such as length of hospital stay, total hospitalization expenses, operation rate, mortality, organ complications, ICU stay, and respirator use exhibited no significant differences between the two groups.

Paralytic ileus may cause endogenous bacterial overgrowth and increased gastrointestinal permeability and intestinal ischemia, and promote bacterial translocation from the intestine, thus resulting in systemic inflammatory response syndrome, secondary infections, sepsis, multiple organ dysfunction syndrome (MODS), and eventually death [26, 27]. Thus, it is important to shorten the duration of paralytic ileus and recover gastrointestinal function to reduce the risks of infection and mortality.

In our study, the QYD1 and QYD2 formulas effectively decreased the duration of paralytic ileus and reduced the rate of infection. Meanwhile, one participant in the test group died from respiratory failure on day 7 after onset of abdominal pain. There was no evidence of infection during the hospitalization. However, two participants in the control group died as a result of MODS on days 14 and 32 after onset of abdominal pain. Further data showed that one of these two participants had undergone surgery because of peripancreatic infection on day 21 after hospitalization, and died of secondary pulmonary infection, bacillosis, and fungal sepsis. The other participant had fungal sepsis, was transferred to the ICU on day 12, and finally died of pyosepticemia. The effectiveness of QYD1 and QYD2 demonstrated in this study may occur primarily through improving intestinal paralysis, recovering the gastrointestinal function, and reducing the bacterial translocation from the intestine, thereby lowering the infection rate and reducing the risk of mortality. Despite these benefits, the other outcomes examined, such as the length of hospital stay, total hospitalization expenses, operation rate, mortality, organ complications, ICU stay, and respirator use exhibited no significant differences between the two groups, although they generally tended to show decreasing trends.

In this study, hyperlipidemia was observed in nearly half of the study subjects, followed by gallstones, alcohol, and idiopathic factors in each group. This situation was different from current findings that the most common causes of AP are gallstones and alcohol [28]. In China, gallstones remain the leading etiological factor for AP, but the incidence of hypertriglyceridemia is increasing [29]. Our results might arise through the eating habits of the individuals who were included in the study. Most of our participants came from Sichuan Province, where people mainly eat the Sichuan cuisine, a diet notable in Chinese cuisine for spicy, pungent, and greasy food. This high-fat diet could easily result in hyperlipidemia.

The most important problem of this study was the definition of SAP. This study used the 2007 Guidelines for Management of SAP in China. The 2007 Guidelines for Management of Severe Acute Pancreatitis in China [25] was the latest definition at that time. The Atlanta Classification of AP was revised in 2012 to provide a more comprehensive classification of severity and peripancreatic conditions [30]. The recruitment of participants was completed in July 2010. We spent more than one year on analyzing the data, writing and revising the manuscript. In the future, we could re-analyze the data according to the latest guideline retrospectively. Furthermore, owing to the limitations of the sample size, this study was unable to determine the precise influences of Qing-Yi Decoction on the length of hospital stay, total hospitalization expenses, operation rate, mortality, organ complications, ICU stay, and respirator use, especially the influence on the incidence of surgery and mortality.

## Conclusions

QYD could restore gastrointestinal motility to normal and reduce the infection rates in the SAP patients who completed a full course of QYD treatment according to per protocol analysis.

## Additional files

Additional file 1:
**Ethical approval of the research protocol.**
Additional file 2:
**Ethical approval of the research protocol.**
Additional file 3:
**Written medical informed consent of participants.**
Additional file 4:
**Questionnaire of follow-up evaluation.**
Additional file 5:
**Intention-to-treat analysis.**

## Abbreviations

SAP: Severe acute pancreatitis
AP: Acute pancreatitis
CM: Chinese medicine
CT: Computerised tomographic
APACHE II: Acute Physiology and Chronic Health Evaluation II
QYD: *Qing-Yi decoction*
QYD1: Num.1 *Qing-Yi decoction*
QYD2: Num.2 *Qing-Yi decoction*
ARF: Acute renal failure
ICU: Intensive care unit
SD: Standard deviation
IAH: Intra-abdominal hypertension
ACS: Abdominal compartment syndrome
SIRS: Systemic inflammatory response syndrome
MODS: Multiple organ dysfunction syndrome
CQCQD: *Chai-Qin-Cheng-Qi Decoction*
DCQD: *Da-Cheng-Qi Decoction*
