# Supplementary material for: Qing-Yi decoction in participants with severe acute pancreatitis: a randomized controlled trial
Source: Chin Med. 2015 May 19;10:11. doi: 10.1186/s13020-015-0039-8 (PMC4449590; doi:10.1186/s13020-015-0039-8)
Supplement: Additional file 1: — Ethical approval of the research protocol. [file 13020_2015_39_MOESM1_ESM.pdf]

# 华西医院生物医学研究项目伦理审批件

2008年 审（12）号

| | | |
|---|---|---|
| 科室/专业：中西医结合科 | | 负责人：夏庆 教授 |
| 项目名称：重症急性胰腺炎中西医结合治疗疗效评价研究 | | |
| 送审方案 | 版本号： 无 | 版本日期：无 |
| 送审知情同意书 | 版本号： 无 | 版本日期：无 |

审查意见：

1、研究者的资格、经验符合研究要求；

2、中药处方改组后新的临床使用时，是否应有药监部门批件；

3、中药处方改组后新的临床使用时，除前期做急性毒性试验外，是否还需做其他基础试验研究后方可做临床试验？

4、方案和知情同意书应在多方面做补充：如增加使用药物的毒副作用，可能的不良反应及不良事件的描述；增加相关保密、风险效益分析、不良事件及损害的补偿、相关费用及免费申明，以及出现不良事件和严重不良事件的处理及处理预案（救治预案等的描述）仅声明不额外增加医疗费用在伦理上是不够的；

5、若先前已做了大量临床研究，则需在方案及受试者须知中简述；

6、建议将受试者须知和知情同意书分开写，并梳理格式，充实内容，更改文字描述，如统一用“受试者”字样，去掉“病友及家属”字样；

7、请提供天津南开医院伦理委员会审批书。

结论：作必要修正后同意。

四川大学华西医院 5101070022002

单位（章）：

主任委员（签名）：[illegible]

2008年 3月11 日
