# Supplementary figures and images for: Qing-Yi decoction in participants with severe acute pancreatitis: a randomized controlled trial
Source: Chin Med. 2015 May 19;10:11. doi: 10.1186/s13020-015-0039-8 (PMC4449590; doi:10.1186/s13020-015-0039-8)

夏庆教授:

收到关于“重症急性胰腺炎中西医结合治疗疗效评价研究”知情同意书修改件和伦理批件的答复。

经评审，同意并予以备案。

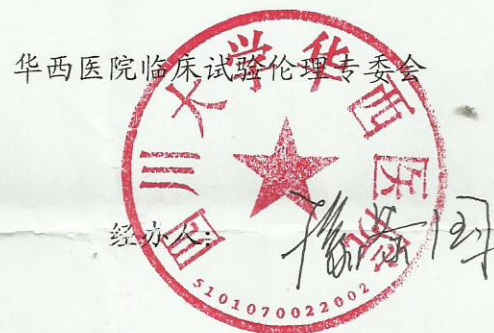

2008年4月1日

Supplement: Additional file 2: — Ethical approval of the research protocol. [file 13020_2015_39_MOESM2_ESM.pdf]
