# Supplementary material for: Qing-Yi decoction in participants with severe acute pancreatitis: a randomized controlled trial
Source: Chin Med. 2015 May 19;10:11. doi: 10.1186/s13020-015-0039-8 (PMC4449590; doi:10.1186/s13020-015-0039-8)
Supplement: Additional file 3: — Written medical informed consent of participants. [file 13020_2015_39_MOESM3_ESM.doc]

中西医结合治疗重症急性胰腺炎疗效及卫生经济学评价

**受试者须知**

亲爱的先生/女士：

您当前所患的疾病为重症急性胰腺炎（SAP），这是一种病死率极高的疾病，一般可达到30－50％，至今全世界范围内都缺乏确切有效的治疗方法。

为了攻克这一医学难题，国家已将“重症急性胰腺炎的中西医结合治疗”列为国家十一五科技支撑计划及国家中医药管理局重点专科专病建设项目，并经过专家严格论证，审核和通过了一系列诊断和治疗的方案。这些方案都是已经经历了大量实验和临床研究，被证实是有一定疗效的，但仍然需要更多的病例进一步验证才能被确认为一种适于全国推广的治疗规范。本院长期致力于SAP治疗的研究工作，具有丰富的经验，因此被选择承担这项重大医学任务。而您也会有机会获得更多有效的治疗药物和手段。

就此，有几点情况应该向您说明：

1．对您的诊断与治疗措施均是依照您的病情需要安排的。因此，对您的诊疗安排与不参加者无区别，在正规性方面甚至要求更严格。

2．您有可能分配在中西医结合治疗组也有可能分配在常规西医治疗组，但治疗措施都是严谨和成熟的。

3．您不会因为参加研究而额外增加不必要的医疗费用。研究中所用试验药物将免费。

4．所有治疗药物都有可能产生副作用。如果在研究中您出现任何不适，或发生新的病情变化，或任何意外情况，不管是否与药物有关，应及时通知您的医生，他/她将对此作出判断和相应医疗处理。如果在临床研究中出现不良事件，医学专家委员会将会鉴定其是否与药物有关。

5．您参与本项目完全是自愿的，您无须任何理由可拒绝参加或中途退出。即使您拒绝参加或中途退出，也不会影响您和医生的关系，更不会受到歧视和报复。

6．您的个人病例资料将完整的保存在医院，您参加本研究及相关的研究资料均属您的个人隐私，我们将在法律允许的范围内严格保密。任何有关本研究结果的公开报告将不会披露您的个人身份。

在本研究的任何阶段，如果您有任何的问题，请您与本院的 医生联系，电话 。

若您理解并同意以上事项，请签字。

谢谢您的合作。

**知情同意书**

我是在仔细阅读知情同意书，并且我的医生给我作了完整的解释后自愿参加这项研究的，我可以在任何时候因任何原因退出本研究。

参加者签字 年 月 日

监护人签字 年 月 日

研究者签字 年 月 日
