# Supplementary material for: Qing-Yi decoction in participants with severe acute pancreatitis: a randomized controlled trial
Source: Chin Med. 2015 May 19;10:11. doi: 10.1186/s13020-015-0039-8 (PMC4449590; doi:10.1186/s13020-015-0039-8)
Supplement: Additional file 4: — Questionnaire of follow-up evaluation. [file 13020_2015_39_MOESM4_ESM.docx]

| 重症急性胰腺炎中西医结合治疗疗效评价研究  课题编号：2006BAI04A15 | 患者姓名： | | 第__次随访 |
| --- | --- | --- | --- |
|  | 药物编号 □□□ | 随访日期  200\|__\|/\|__\|__\|/\|__\|__\| |  |

| 【注释】实验室检查结果的临床意义判断，按如下标准进行：①正常；②异常、但无临床意义；  ③异常、有临床意义；④未查。请选择相应框内划“×”。 |
| --- |

**一、基础检查：**

| 检查项目 | | 检查结果 | 单位 | 检查结果临床意义的判断 | | | |
| --- | --- | --- | --- | --- | --- | --- | --- |
| 体  格  检  查 | 体温 | □□.□ | ℃ | □① | □② | □③ | □④ |
|  | 心率 | □□□ | 次/分 | □① | □② | □③ | □④ |
|  | 呼吸 | □□ | 次/分 | □① | □② | □③ | □④ |
|  | 收缩压 | □□□ | mmHg | □① | □② | □③ | □④ |
|  | 舒张压 | □□□ | mmHg | □① | □② | □③ | □④ |
| 实  验  室  检  查 | 红细胞压积 | □□.□ | % | □① | □② | □③ | □④ |
|  | 血白细胞计数 | □□.□ | 10^9/L | □① | □② | □③ | □④ |
|  | 血钠 | □□□ | mmol/L | □① | □② | □③ | □④ |
|  | 血钾 | □.□□ | mmol/L | □① | □② | □③ | □④ |
| 生  化  检  查 | 总胆红素（TB） | □□□ | umol/L | □① | □② | □③ | □④ |
|  | 谷草转氨酶（AST） | □□□ | U/L | □① | □② | □③ | □④ |
|  | 谷丙转氨酶（ALT） | □□□ | U/L | □① | □② | □③ | □④ |
|  | 尿素氮（BUN） | □□.□ | mmol//L | □① | □② | □③ | □④ |
|  | 肌酐（CRE） | □□□ | umol/L | □① | □② | □③ | □④ |
|  | 葡萄糖（GLU） | □.□□ | mmol/L | □① | □② | □③ | □④ |
|  | 胆固醇（CHOL） | □□.□ | mmol//L | □① | □② | □③ | □④ |
|  | 甘油三酯（TG） | □□.□ | mmol//L | □① | □② | □③ | □④ |

**二、常规检查：**

| 检查项目 | | 检查结果 | 单位 | 检查结果临床意义的判断 | | | |
| --- | --- | --- | --- | --- | --- | --- | --- |
| 常规  室检  查 | 血淀粉酶 | □□□ | U/L | □① | □② | □③ | □④ |
|  | 尿淀粉酶 | □□□□ | U/L | □① | □② | □③ | □④ |
|  | 血脂肪酶 | □□□ | U/L | □① | □② | □③ | □④ |
|  | 血游离钙 | □.□□ | mg/L | □① | □② | □③ | □④ |

观察医师签名□□□□ 日期□□□□/□□/□□

**三、腹部CT：**

| 腹部CT结果： | Balthartz评分： |
| --- | --- |

**四、中医症候：**

| **腹痛** |
| --- |
| □1.腹痛拘急，遇冷痛剧 □2.腹痛急迫，痛处灼热  □3.腹痛胀满，痛处不定，攻撑作痛，痛引两胁 □4.腹部刺痛，痛处不移，拒按  □5.脘腹胀满，嗳气频作，嗳腐吞酸 □6.腹痛隐隐，喜揉喜按  □7. 无腹痛 □8.其他： |
| **腹胀** |
| □1.胃脘痞满，灼热急迫 □2.脘腹满闷，痞塞不舒，嗳腐吞酸  □3.脘腹不舒，胸胁胀满，心烦易怒 □4. 脘腹痞闷，喜温喜按，疲倦乏力  □5.无腹胀 □6．其他： |
| **大便** |
| □1.粪质干燥坚硬，肛门灼热 □2. 粪质干结，如羊屎状  □3.粪质干结，面色无华 □4. 粪质不干，欲便不出，便下无力  □5.粪质不甚干结，排出断续不畅 □6．便稀，小便清长，面色晄白，四肢不温  □7.大便正常 □8．其他： |
| **寒热** |
| □1.壮热，体温39度以上，持续不退 □2.日脯潮热，下午3点到五点发热明显  □3.午后发热，身热不扬 □4.夜间潮热，五心烦热  □5.发热不高，体温不超过38度 □6.寒战与高热交替发作  □7.畏寒肢冷，喜温 □8.无寒热  □9.其他： |
| **舌质** |
| □1.淡红 □2.淡白 □3.红绛 □4.青紫 □5.紫黯  □6.胖大 □7. 瘦薄 □8.裂纹  □9.其他： |
| **舌苔** |
| □1.白苔 □2.黄苔 □3.灰黑苔  □4.薄 □5.厚 □6.润 □7.燥  □8.腻苔 □9. 腐苔 □10.剥苔 □11.其他： |

观察医师签名□□□□ 日期□□□□/□□/□□
