# Supplementary material for: Qing-Yi decoction in participants with severe acute pancreatitis: a randomized controlled trial
Source: Chin Med. 2015 May 19;10:11. doi: 10.1186/s13020-015-0039-8 (PMC4449590; doi:10.1186/s13020-015-0039-8)
Supplement: Additional file 5: — Intention-to-treat analysis. [file 13020_2015_39_MOESM5_ESM.docx]

**Table S1** Demographics and clinical characteristics of participants

|  | Test group (*n*=50) | Control group (*n*=50) | *P* value |
| --- | --- | --- | --- |
| **Age (mean ± SD) ^†^**  years | 46.48 (11.15) | 45.96 (9.69) | 0.804 |
| **Sex, *n* (%) ^‡^** |  |  | 0.688 |
| Male | 28 (44) | 26 (52) |  |
| Female | 22 (56) | 24 (48) |  |
| **Etiology, *n* (%) ^‡^** |  |  | 0.259 |
| Biliary | 12 (24) | 12 (24) |  |
| Hyperlipidemic | 28 (56) | 20 (40) |  |
| Alcoholic | 4 (8) | 5 (10) |  |
| Idiopathic | 6 (12) | 13 (26) |  |
| **Scores (median (interquartile range)) ^§^**  Ranson’s score | 3 (3-5) | 4 (3-5) | 0.653 |
| 24 h APACHE II score | 10 (8-13) | 9 (5.8-13) | 0.251 |
| Balthazar CT score | 4 (4-6) | 4 (4-6) | 0.160 |

^†^ Two independent samples *t*-test, ^‡^ Chi-square test, ^§^Wilcoxon rank sum test.

APACHE II: Acute Physiology and Chronic Health Evaluation II

**Table S2** Primary end points

|  | Test group (*n*=50) | Control group (*n*=50) | *P* value |
| --- | --- | --- | --- |
| Hospital stay, days (median (interquartile range) ^§^) | 20 (14.8-27) | 20 (14-28) | 0.817 |
| Total hospitalization expenses (RMB; median (interquartile range) ^§^) | 33330.5 (24311.7-44989.0) | 37490.1 (23765.9-64094.6) | 0.356 |
| Operation, *n* (%) ^‡^ | 1(2) | 3(6) | 0.307 |
| Mortality, *n* (%) ^‡^ | 1(2) | 2(4) | 0.558 |

^‡^ Chi-square test, ^§^Wilcoxon rank sum test.

**Table S3** Secondary end points

|  | Test group (*n*=50) | Control group (*n*=50) | *P* value |
| --- | --- | --- | --- |
| Organ complications |  |  |  |
| Heart failure, *n* (%) ^‡^  Duration of heart failure, days  median (interquartile range) ^§^  Respiratory failure, *n* (%) ^‡^ | 13 (26)  0 (0-1)  30 (60) | 15 (30)  0 (0-2)  29 (58) | 0.656  0.448  0.839 |
| Duration of respiratory failure, days median (interquartile range) ^§^ | 1 (0-3) | 2 (0-4) | 0.521 |
| ARF, *n* (%) ^‡^ | 5 (10) | 7 (14) | 0.538 |
| Duration of ARF, days  median (interquartile range) ^§^ | 0 (0-0) | 0 (0-0) | 0.471 |
| Hepatic failure, *n* (%) ^‡^ | 11 (22) | 16 (32) | 0.260 |
| Duration of hepatic failure, days  median (interquartile range) ^§^ | 0 (0-0) | 0 (0-2.3) | 0.153 |
| Paralytic ileus, *n* (%) ^‡^ | 47 (94) | 43 (86) | 0.182 |
| Duration of paralytic ileus, days  median (interquartile range) ^§^ | 4 (2-6)^a^ | 6 (4-8.3) | 0.020 |
| Infection, *n* (%) ^‡^ | 7 (14)^a^ | 16 (32) | 0.032 |
| ICU stay, days  median (interquartile range) ^§^ | 0 (0-0) | 0 (0-0.5) | 0.126 |
| Use of respirator, *n* (%) ^‡^ | 6 (12) | 13 (26) | 0.074 |
| Use of respirator, days  median (interquartile range) ^§^ | 0 (0-0) | 0 (0-2.3) | 0.072 |

^‡^ Chi-square test, ^§^ Wilcoxon rank sum test. ^a^ *P*<0.05 vs. control group.

ARF: acute renal failure

ICU: intensive care unit
